# Biobased Ionic Liquids as Multitalented Materials in Lipidic Drug Implants

**DOI:** 10.3390/pharmaceutics13081163

**Published:** 2021-07-28

**Authors:** Ana Júlio, Anaisa Sultane, Ana Silveira Viana, Joana Portugal Mota, Tânia Santos de Almeida

**Affiliations:** 1 CBIOS-Universidade Lusófona’s Research Center for Biosciences & Health Technologies, Campo Grande 376, 1749-024 Lisboa, Portugal; ana.julio@ulusofona.pt (A.J.); asultane@gmail.com (A.S.); joana.mota@ulusofona.pt (J.P.M.); 2 Department of Biomedical Sciences, University of Alcalá, Ctra. Madrid-Barcelona Km. 33.600, Alcalá de Henares, 28871 Madrid, Spain; 3 Centro de Química Estrutural, Faculdade de Ciências, Universidade de Lisboa, Campo Grande, 1749-016 Lisbon, Portugal; anaviana@fc.ul.pt

**Keywords:** lipidic implants, caffeine, salicylic acid, rutin, ionic liquids, improved performance

## Abstract

Lipidic implants are valuable controlled delivery systems that present good biocompatibility and are useful for long-lasting therapies. However, these promising systems can present inflexible drug release profiles that limit their performance. Thus, finding new materials to overcome this drawback is crucial. Herein, lipidic implants containing caffeine and poorly soluble salicylic acid and rutin were developed. The inclusion of Gelucire^®^ 50/02, sucrose, and two biobased ionic liquids, [Cho][Phe] and [Cho][Glu], were evaluated as a mean to improve the performance of the systems. The formulation procedure, dye content distribution, drug content, drug release, water content, and lipidic erosion of the developed systems were assessed. AFM analysis of the implants containing ILs was also performed. The results demonstrated that neither Gelucire^®^ 50/02 nor sucrose were suitable tools to improve the drug release profile. In contrast, the ILs proved to be promising materials for multiple reasons; not only did they facilitate the formulation and incorporation of the studied drugs into the implants, but they also allowed a more suitable release profile, with [Cho][Glu] allowing a higher drug release due to its ability to increase surface wrinkling. Hence, this study showcases ILs as multitalented materials in lipid-based drug implants.

## 1. Introduction

The pharmaceutical industry has always sought to develop drug delivery systems that allow for a prolonged and effective therapeutic effect to ensure less adverse effects and less frequency of administration. Among these controlled delivery systems are implants that are particularly useful in prolonged therapies [1,2]. These systems are inserted subcutaneously (into the interstitial tissues of the arm, thigh, or abdomen) when a systemic effect is sought. In contrast, if a localized effect is desired, they may be placed in the target organ through surgical procedures (for example, in the vitreous cavity of the eye or intraperitoneally) [3].

Moreover, there are three types of implants including polymeric, mini-pumps, or lipidic. The polymeric implants have different shapes and are composed of biodegradable or non-biodegradable polymers, while mini-pumps are prepared by combining a polymer and titanium, thus containing an osmotic system [3,4,5,6]. In contrast, the lipidic implants are produced with lipids that contribute to a higher biocompatibility and low toxicity compared to polymeric implants [3,4,5,6]. Hence, there are various studies focused on lipidic implants due to their attractive properties compared to polymeric ones [3,4,5,6]. Apart from their low toxicity and good biocompatibility, due to the presence of lipids that are constituents of the organism, their ability for drug protection and flexibility in choosing different excipients in the formulation process also represent advantages [3,4,5,6,7,8]. However, they may present inflexible drug release profiles such as incomplete total drug release that then leads to a low efficiency of the system [3,6]. Additionally, they may also present some issues concerning degradation [3,6,9].

Hence, when developing new implants, it should be considered that while the lipidic implants are biocompatible and have low toxicity, these delivery systems must also be prepared through a straightforward and efficient method that allows for uniform drug distribution and leads to the desired drug release profile [3,5,6,10]. To achieve these goals, the type of materials selected to be incorporated in the implants may be crucial. Considering this, ionic liquids (ILs) were used in this study to evaluate their possible applicability as multifunctional components in lipidic implants.

ILs are poorly coordinated organic salts that have been defined as a liquid below 100 °C or even at room temperature [11,12,13,14,15]. They have high thermal and chemical stability, low vapor pressure, are negligibly volatile, and present good ionic conductivity [16,17]. ILs have been used in pharmaceutical research for several applications [18] such as solubility promotors [12,13,15,19,20,21,22], as catalysts in the synthesis of active pharmaceutical ingredients [19,23,24], as oil or water substitutes [24,25,26,27], as surfactants in emulsions and micro emulsions [12,15,19,20,25,26,27], or even integrated with nanomaterials [28,29,30,31]. Hence, ILs may potentially be key materials to be incorporated in different delivery systems such as lipidic implants to improve their efficiency. Specifically in this study, two choline-based ILs were incorporated into lipidic implants at non-toxic concentrations [13,15] and the impact of these materials on the formulation procedure and in the performance of the developed systems was evaluated. The lipidic implants were produced in the presence and absence of ILs with variable compositions and with or without the studied drugs, namely caffeine and poorly water-soluble salicylic acid and rutin.

The three incorporated drugs were chosen as they each have pharmaceutical interest. Caffeine, a natural methylxanthine alkaloid, salicylic acid, a natural β-hydroxy acid, and rutin, a polyphenolic bioflavonoid, each have activity at the level of the central nervous system which may be useful in the treatment of some neurodegenerative diseases as a stimulant [32,33,34,35], anticonvulsant [35,36,37,38], sedative [35,36,39], and possibly as a suppressor of the neurological pathway that contributes to the onset of tardive dyskinesia, common in Alzheimer’s and Parkinson’s [35,36,37,38]. In the case of neurodegenerative diseases, the application of lipidic implants for the purpose of achieving a controlled delivery may be an added value by allowing for a higher adherence to therapy and reducing the number of doses, while decreasing plasma oscillations of drug concentrations and avoiding toxic levels [40,41,42]. Thus, due to the importance that lipidic implants may represent in terms of controlled delivery and the relevance of finding resourceful and multi-talented materials that may improve the efficiency of these systems, herein, ionic liquids were incorporated into lipidic implants. The outcome of this inclusion was evaluated in multiple ways. To achieve this, several implants were developed, and the impact of the different compositions were studied including: (1) the efficiency of the development procedure; (2) the distribution of different dyes; (3) the drug content; (4) the water content; (5) the lipidic erosion; and (6) the drug release profile.

## 2. Materials and Methods

### 2.1. Materials and Reagents

The lipidic implants were produced using Dynasan^®^ 118; glyceryl tristearate from Cremer Oleo GmbH (Hamburg, Germany); Gelucire^®^ 50/02 which is a mixture of glycerol monoesters, diesters, and tri-esters with polyethylene glycol monoesters and diesters from GatteFossé (Saint-Priest, France); and sucrose from Sigma-Aldrich (Darmstadt, Germany). The chosen drugs were, caffeine and salicylic acid, both from Sigma-Aldrich (St. Louis, MO, USA), and rutin from Fagron (São Paulo, Brazil). To evaluate the dye content distribution, the developed lipidic implants were produced in the presence of one lipophilic dye, Sudan III, or one hydrophilic dye, Methylene Blue, purchased from Sigma-Aldrich (St. Louis, MO, USA) and Sigma-Aldrich (Darmstadt, Germany), respectively. The incorporated ILs, namely (2-hydroxyethyl)-trimethylammonium-l-phenylalaninate [Cho][Phe] and (2-hydroxyethyl)-trimethylammonium-l-glutaminate [Cho][Glu] were synthesized and characterized within the context of other studies developed by our group [12,15].

For the drug release, water content, and the lipidic erosion studies, phosphate-buffered saline (PBS) pH 7.4 was used and prepared in house with 0.01% (*w*/*w*) sodium azide from Sigma-Aldrich (St. Louis, MO, USA) used as release medium. This medium was also used to assess the drug content and both the diethyl ether from PanReac AppliChem^®^ ITW Reagents (Barcelona, Spain) and the ethanol absolute from Sigma-Aldrich (St. Louis, MO, USA). As equipment, a Heidolph 1000^®^ incubator with a stirring Heidolph Unimax 1010^®^ (Schwabach, Germany), a multipoint plate IKA^®^ RT 15P (Staufen, Germany), and an Evolution^®^ spectrophotometer (Thermo Scientific, Hertfordshire, England) was used.

### 2.2. Implants Preparation

The lipidic implants were prepared manually by fusion and melting through a technique modified from the method previously described by Kreye et al. [3] with the aim of improving the preparation procedure and reducing material loss. Firstly, the drug was sprayed and sieved with a diameter under 100 µm, before being used. Then, the lipid (Dynasan^®^ 118), each release adjuvant of sucrose or Gelucire^®^ 50/02 (the first value denotes the melting point of the substance and the second the value notes the hydrophilic lipophilic balance-HLB), and/or the IL used were weighed.

The drug was also weighed and added to the respective mixtures. All mixtures were heated with stirring (100 rpm) in a water bath at 80 °C in the heating and stirring plate IKA^®^ 45 (Staufen, Germany) until the melting, complete fusion, and homogenization (with stirring) of the samples was achieved. The fused mixtures were pipetted with a sterile disposable cylinder. All batches presented a total batch weight of 3 g. After cooling to room temperature, the implants were removed from the containers and stored under moisture-free conditions. Prior to being used, the implants were cut to equally determined sizes (0.5 cm). For the implants containing the studied drugs, the lipid:drug ratio was 90:10% (*w*/*w*). The composition of all prepared implants is described in Table 1. The total weight of the prepared implants range between 26 mg to 29 mg and the drug content and release studies were performed based on the total weight of each implant.

### 2.3. Dye Content Distribution

To assess the distribution of the two dyes, the lipidic implants were produced as previously described but now also containing a lipophilic or hydrophilic dye solution of 2.5% *w*/*w* of Sudan III [43] or Methylene Blue [44] (Table 1).

### 2.4. Drug Content

#### 2.4.1. Implants Containing Caffeine

The samples (*n* = 3) containing caffeine were dissolved in a mixture of 1.5 mL of PBS pH 7.4 with 0.01% (*w*/*w*) of sodium azide and 0.5 mL of diethyl ether and were then placed under stirring at 37 °C in the multipoint plate IKA^®^ RT 15P (Staufen, Germany). After 90 min and 180 min, an aliquot of the aqueous phase (100 μL) was removed and quantified using an Evolution^®^ spectrophotometer (Thermo Scientific, Hertfordshire, England) at 273 nm (maximum absorption wavelength in PBS solution). The percentage of the drug was calculated based on the total weight of each implant.

#### 2.4.2. Implants Containing Salicylic Acid or Rutin

Implants (*n* = 3) were crushed and dispersed in 25 mL of absolute ethanol. The drug (salicylic acid or rutin) was completely dissolved, whereas implant excipients were dispersed. An aliquot (1 mL) was filtered, diluted, and then the drug quantification was performed by UV-Vis spectrophotometry (Evolution^®^ spectrophotometer, Thermo Scientific, Hertfordshire, England) at the maximum absorption wavelength for each drug (295 nm for salicylic acid or 348 nm for rutin) in absolute ethanol. The drug content was calculated based on the total weight of each implant.

### 2.5. In Vitro Drug Release

The implants (*n* = 5) were placed in 1.5 mL PBS pH 7.4 with 0.01% (*w*/*w*) of sodium azide at 37 °C and 100 rpm in a Heidolph 1000^®^ incubator with stirring (Heidolph Unimax 1010^®^, Schwabach, Germany). At predetermined time points, the drug release was measured by UV-Vis spectrophotometry in an Evolution^®^ spectrophotometer (Thermo Scientific, Hertfordshire, England) at the maximum absorption wavelength of each drug in PBS (273 nm for caffeine, 281 nm for salicylic acid, and 354 nm for rutin). To achieve this, after removing the totality of the release medium for analysis, this medium was always completely replaced with fresh PBS pH 7.4 (1.5 mL). This study was performed for 140 days as follows. In the first week, the samples were analyzed every day. In the second and third weeks, these analyses were performed biweekly. In the following days the measurements were done once a week. Sink conditions were kept in all experiments throughout the study. Once again, the percentage of drug release was obtained considering the total weight of each implant.

### 2.6. Water Content and Lipidic Erosion

The implants (*n* = 5) were weighed [dry mass (*t* = 0)] and placed in 1.5 mL PBS pH 7.4 with 0.01% (*w*/*w*) of sodium azide at 37 °C and 100 rpm in a Heidolph 1000^®^ incubator with stirring (Heidolph Unimax 1010^®^, Schwabach, Germany).

At the same predetermined time points of the in vitro release studies, the implants were removed from the medium and carefully dried so that the droplets of medium on the surface were removed. Then, the implants were weighed, obtaining the wet mass (*t*), and then dried at 37 °C in an oven (Memmert U30^®^ from Memmert, Schwabach, Germany) until a constant mass was obtained which was designated as dry mass (*t*). The release medium was analyzed by UV-Vis spectrophotometry in an Evolution^®^ spectrophotometer (Thermo Scientific, Hertfordshire, England) at the maximum absorption wavelength of each drug in PBS to measure the drug released at time *t*.

The water content (*WC*) and the lipidic erosion (*LE*), both in percentage (%), were calculated by the following equations:

(1) $$WC\;\left(\%\right)=\frac{wet\;mass\;\left(t\right)-dry\;mass\;\left(t\right)}{wet\;mass\left(t\right)}\times 100$$

(2) $$LE\;\left(\%\right)=\frac{dry\;mass\;\left(t=0\right)-drug\;released\;\left(t\right)-dry\;mass\left(t\right)}{dry\;mass\left(0\right)}\times 100$$

### 2.7. Atomic Force Microscopy (AFM)

AFM measurements were conducted in air (23 ± 1 °C) on a Multimode 8HR microscope coupled to a Nanoscope V (Bruker Corporation, Billerica, MA, USA). The images were acquired in tapping mode using etched silicon probes with a resonance frequency of ca. 75 kHz (FESP, Bruker Corporation, Billerica, MA, USA) and at a scan rate of ~1.3 Hz.

As the implants were previously cut with 0.5 cm, the samples were then sectioned half-longitudinally in length and glued directly onto the AFM magnetic specimens for imaging. At least two regions of each sample were imaged.

### 2.8. Statistical Analysis

After conducting normality and homogeneity tests, the results were expressed as mean ± standard deviation (SD) and evaluated with the Kruskal–Wallis test, followed by Bonferroni correction test or one-way analysis of variance (ANOVA) and then by Tukey’s multiple comparison test. The differences between individual means were significant at * *p* < 0.05, ** *p* < 0.01, and *** *p* < 0.001. The analyses were performed using the SPSS^®^ statistical package (version 25, SPSS Inc. Chicago, IL, USA).

## 3. Results and Discussion

Several lipidic implants with different compositions, namely different release adjuvants (Gelucire^®^ 50/02 or sucrose), and in the presence or absence of two biobased ILs derived from natural amino acids ([Cho][Phe] or [Cho][Glu]) were prepared. This was performed to assess the impact of the incorporated excipients, particularly the studied ILs, on the performance of the developed delivery systems and to establish if these materials could exhibit several functionalities.

It should be noted that to prove the ILs actually have valid functionalities, these materials were incorporated into the formulations at non-toxic concentrations, namely 0.2% (*v*/*v*) that is known to be the maximum percentage where cell viability is maintained in HaCaT cells (human keratinocytes) from previous studies performed by our group [13,15].

One of the initial goals of this study was to improve the implants’ preparation technique as it is crucial to have a simple and effective method that allows to attain uniform implants. Hence, a modified melting and fusion method that uses a single container mold was developed. This allowed for a better distribution of the various components of the prepared delivery systems and led not only to a faster production of the implants but also to a reduction in material loss, decreasing the production cost.

### 3.1. Dye Content Distribution

**Figure 1:**
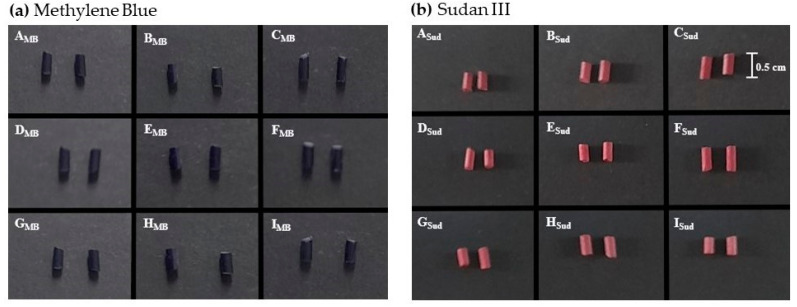
Macroscopic appearance of the developed implants containing (**a**) the hydrophilic methylene blue (**A_MB_–I_MB_**) or (**b**) the lipophilic sudan III (**A_Sud_–I_Sud_**) dyes in the distribution assessment.

Before incorporating the three studied drugs, all implants were prepared in the presence of a hydrophilic (Methylene blue-**a**) or a lipophilic (Sudan III-**b**) dye (Table 1). This was performed to evaluate whether the developed preparation procedure and chosen excipients including the ILs would allow for an even distribution of both model dyes and then to infer if we could effectively incorporate both hydrophilic and lipophilic drugs into the developed implants. In fact, the results revealed (Figure 1) that all batches either containing the dyes of **a** or **b** presented a homogeneous appearance on the surface and on the cross section of the implants, suggesting that the preparation technique performed under the studied compositions leads to a uniform distribution.

Moreover, slight differences in color intensity between different batches were only observed due to the presence of different components that contribute differently to the observed shades. For instance, the implants containing the ILs presented a more intense color. This was not surprising considering the studied ILs are slightly colored.

After demonstrating that the used methodology and chosen components seem to allow a uniform distribution of both hydrophilic and lipophilic compounds, it was justified to move towards the incorporation of different drugs in the implants.

### 3.2. Implants Containing Each Drugs

**Figure 2:**
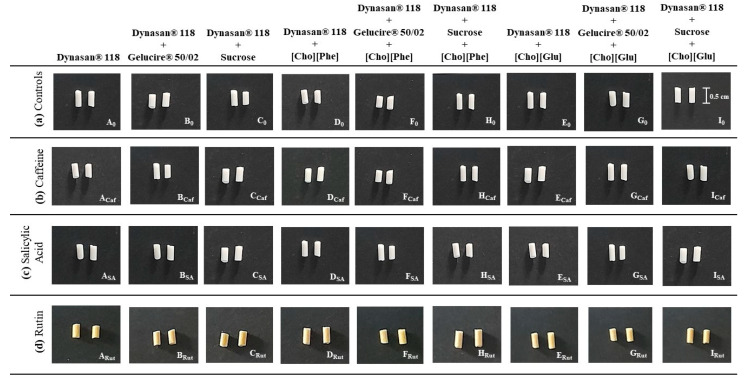
Macroscopic appearance of all prepared implants: without drug (**A_0_–I_0_**), containing caffeine (**A_Caf_–I_Caf_**), salicylic acid (**A_SA_–I_SA_**), or rutin (**A_Rut_–I_Rut_**).

The lipidic systems were then prepared in the presence of the three studied drugs, namely caffeine, a more hydrophilic active, and salicylic acid or rutin, both more lipophilic drugs compared to caffeine (Table 1, Figure 2).

It should be noted that the incorporation of the studied drugs was facilitated for the implants containing ILs when compared to the implants without ILs. This may be due to the fact that these salts are known to be good solubility promotors [12,13] and consequently they likely allow a better incorporation of the studied drugs as well as a better blend of the different components contained in the studied formulations. These were the first indicators that including ILs into the developed implants could be a valuable strategy to improve the performance of lipid-based formulations.

Furthermore, all implants presented a similar appearance, differing only slightly in color depending on the incorporated drug and/or IL (Figure 2). This reveals once again that all the used excipients do not seem to interfere with the homogeneity of the developed implants.

#### 3.2.1. Drug Content

**Figure 3:**
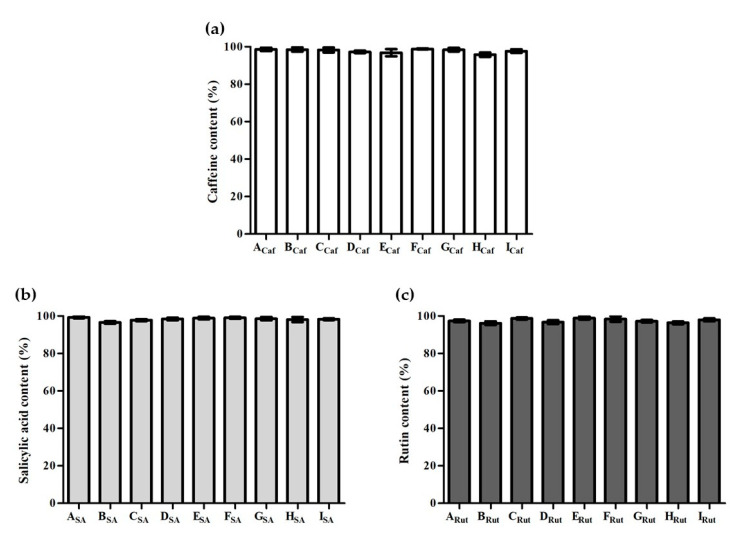
Drug content (%) of the implants containing (**a**) caffeine (**A_Caf_**–**I_Caf_**), (**b**) salicylic acid (**A_SA_**–**I_SA_**), and (**c**) rutin (**A_Rut_**–**I_Rut_**). *n* = 3, mean ± SD.

To further ensure the efficiency of the developed methodology, the percentage of drug content of all the implants containing each studied drug was also evaluated. In this assessment, results showed that the drug content was greater than 95% for all the implants containing each of the three drugs (Figure 3).

No statistical differences were observed between the different formulations. This outcome corroborates the robustness of the methodology used to prepare the implants, demonstrating a uniform drug content.

**Figure 4:**
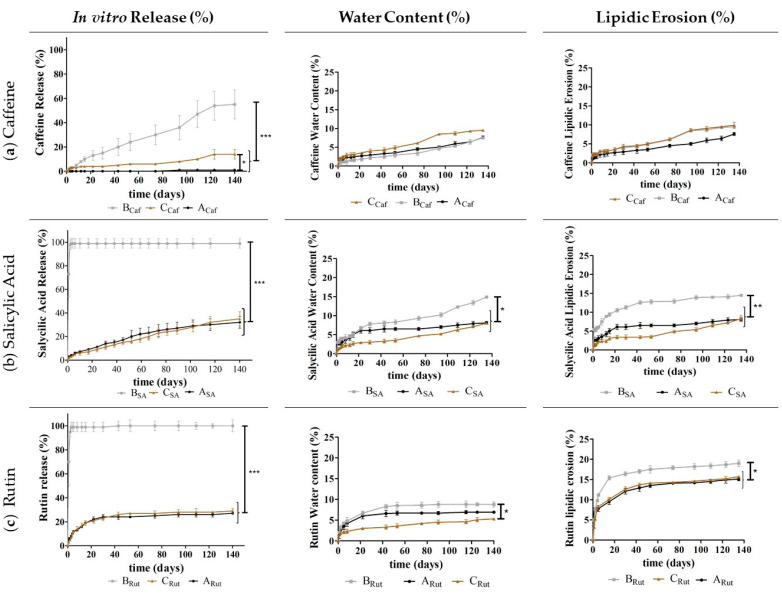
In vitro drug release (%), water content (%), and lipidic erosion (%) of the implants with Dynasan^®^ 118 (**A_Caf_**, **A_SA_**, and **A_Ru_**_t_); Dynasan^®^ 118 and Gelucire^®^ 50/02 (**B_Caf_**, **B_SA_**, and **B_Rut_**); and Dynasan^®^ 118 and sucrose (**C_Caf_**, **C_SA_**, and **C_Rut_**) in the presence of caffeine (**a**), salicylic acid (**b**), or rutin (**c**). *n* = 5, mean ± SD, * *p* < 0.05, ** *p* < 0.01, and *** *p* < 0.001.

**Figure 5:**
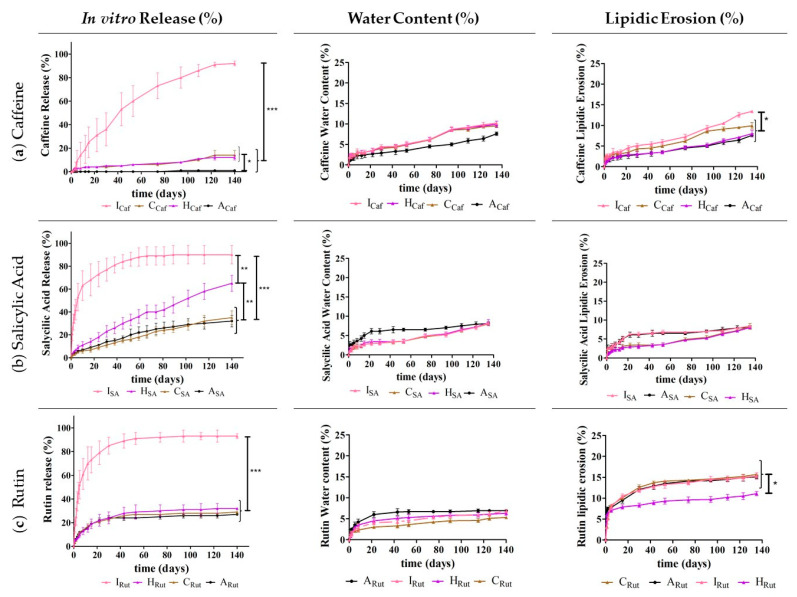
In vitro drug release (%), water content (%), and lipidic erosion (%) of the implants with Dynasan^®^ 118 (**A_Caf_**, **A_SA_**, and **A_Rut_**); with Dynasan^®^ 118 and sucrose (**C_Caf_**, **C_SA_**, and **C_Rut_**); and with Dynasan^®^ 118, sucrose, and the IL [Cho][Phe] (**H_Caf_**, **H_SA_**, and **H_Rut_**) or [Cho][Glu] (**I_Caf_**, **I_SA_**, and **I_Rut_**). Each batch was prepared in the presence of caffeine (**a**), salicylic acid (**b**), and rutin (**c**). *n* = 5, mean ± SD, * *p* < 0.05, ** *p* < 0.01, and *** *p* < 0.001.

**Figure 6:**
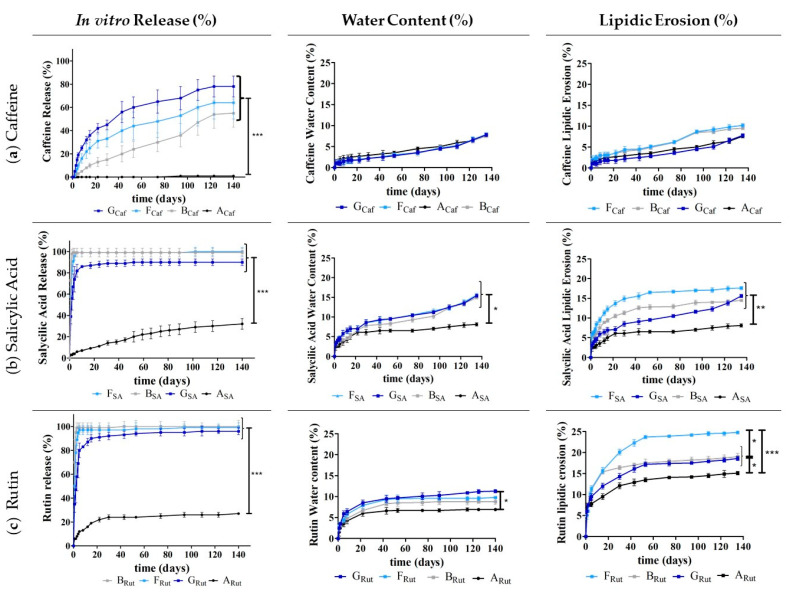
In vitro drug release (%), water content (%), and lipidic erosion (%) of the implants with Dynasan^®^ 118 (**A_Caf_**, **A_SA_**, and **A_Rut_**); with Dynasan^®^ 118 and Gelucire^®^ 50:02 (**B_Caf_**, **B_SA_**, and **B_Rut_**); and with Dynasan^®^ 118, Gelucire^®^ 50:02, and IL [Cho][Phe] (**F_Caf_**, **F_SA_**, and **F_Rut_**) or [Cho][Glu], (**G_Caf_**, **G_SA_**, and **G_Rut_**). Each batch was prepared in the presence of caffeine (**a**), salicylic acid (**b**), and rutin (**c**). *n*=5, mean ± SD, * *p* < 0.05, ** *p* < 0.01 and *** *p* < 0.001.

**Figure 7:**
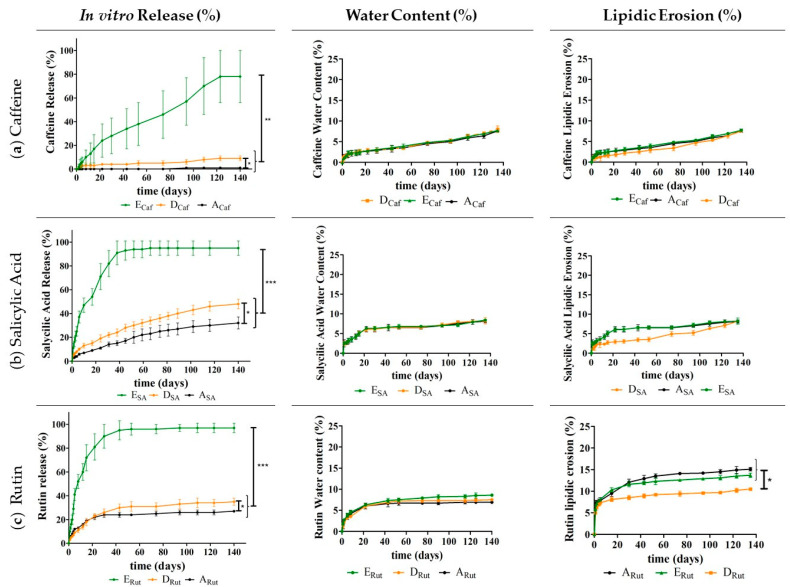
In vitro drug release (%), water content (%), and lipidic erosion (%) of the implants with Dynasan^®^ 118 (**A_Caf_**, **A_SA_**, and **A_Rut_**); Dynasan^®^ 118 and [Cho][Phe] (**D_Caf_**, **D_SA_**, and **D_Rut_**); and Dynasan^®^ 118 and [Cho][Glu] (j**E_Caf_**, **E_SA_**, and **E_Rut_**). Each batch was prepared in the presence of caffeine (**a**), salicylic acid (**b**), and rutin (**c**). *n* = 5, mean ± SD, * *p* < 0.05, ** *p* < 0.01 and *** *p* < 0.001.

Then, to continue to evaluate the performance of the developed implants and the impact of the used release adjuvants (Gelucire^®^ 50/02 or sucrose) and of the two ILs, several studies were implemented, namely studying the in vitro drug release, water content, and lipidic erosion (Figure 4, Figure 5, Figure 6 and Figure 7).

#### 3.2.2. In Vitro Drug Release, Water Content, and Lipidic Erosion

When comparing the inclusion of Gelucire^®^ 50/02 or sucrose in the implants, the drug release results revealed that Gelucire^®^ 50/02 is a better drug release promotor (Figure 4).

This is particularly evident for the implants in the presence of salicylic acid (**B_SA_**) or rutin (**B_Rut_**) for which an immediate drug release was observed for the implants containing Dynasan^®^ 118 and Gelucire^®^ 50/02, in opposition to the lower drug release observed for the implants containing Dynasan^®^ 118 and sucrose (**C_SA_** and **C_Rut_**) or for the implants containing only Dynasan^®^ 118 (**A_SA_** and **A_Rut_**).

With respect to the water content and lipidic erosion, no substantial differences were observed, although for the systems containing salicylic acid and rutin, the implants with Gelucire^®^ 50/02 exhibited a slightly higher lipid erosion compared to the implants with sucrose. Although, this aspect may somewhat contribute to the higher drug release observed, when considering the implants containing Gelucire^®^ 50/02, this difference is not substantial enough to be the sole contributor for the immediate drug release observed for these implants. Conversely, a higher affinity between the two more lipophilic drugs and the more lipophilic carrier Gelucire^®^ 50/02 may facilitate the drug diffusion in this material and thus be a more relevant contributor for the higher drug release.

What is more interesting is that neither Gelucire^®^ 50/02 nor sucrose alone appeared to be the best choice to ensure the desired controlled release over time. For Gelucire^®^ 50/02, a non-desired immediate release was observed for the more lipophilic actives, while with sucrose, even though the release profile was sustained over time, it was quite low after the 140 days, not exceeding a 35% release. For caffeine, a low drug release was observed after the 140 days in the presence of both materials.

Then, we considered the impact of each ionic liquid, [Cho][Phe] or [Cho][Glu], on the performance of the developed implants to assess whether including these materials could be a better strategy.

When evaluating whether combining sucrose (Figure 5) or Gelucire^®^ 50/02 (Figure 6) with each IL could be useful to improve the drug release profile, our results indicated that for caffeine (**a**), both combinations generally allowed for an increase in drug release compared to the implants containing only Dynasan^®^ 118 (Figure 5a and Figure 6a). This increase is more obvious for [Cho][Glu] (Figure 5, **I_Caf_** and Figure 6, **G_Caf_**).

For salicylic acid and rutin, the combination of the IL [Cho][Glu] with either sucrose (Figure 5) or Gelucire^®^ 50/02 (Figure 6) also leads to a higher drug release. Nonetheless, for these compounds, the combination of the ILs ([Cho][Phe] or [Cho][Glu]) with Gelucire^®^ 50/02 (**F_SA_** and **F_Rut_,** and **G_SA_** and **G_Rut_**) led to a similar immediate release as observed for Gelucire^®^ 50/02 alone (**B_SA_** and **B_Rut_**), proving that this combination is not helpful to attain a more controlled and suited release profile.

The next step was to evaluate whether including each IL alone on the implants (**D_Caf_**, **D_SA_**, and **D_Rut_,** and **E_Caf_**, **E_SA_**, and **E_Rut_**) without Sucrose nor Gelucire^®^ 50/02 could be a better approach to improve the performance of the implants (Figure 7).

Compared to the systems containing only Dynasan^®^ 118 (Figure 7, batches **A_Caf_**, **A_SA_,** and **A_Rut_**), the presence of the IL [Cho][Phe] alone led to a slight increase in drug release for the three studied drugs (Figure 7, **D_Caf_**, **D_SA_,** and **D_Rut_**). This release was also superior to what was previously observed in the presence of sucrose (Figure 4). Nonetheless, after the 140 days, the observed drug release was still relatively low for the three drugs (lower than 50%).

It is also important to note that in the presence of [Cho][Phe], the release was controlled over time, demonstrating that this IL allows for a more suited release profile compared to what was observed in the presence of Gelucire^®^ 50/02 (Figure 4). Thus, including [Cho][Phe] alone into the Dynasan^®^ 118 implants seems to be a better strategy then using sucrose or Gelucire^®^ 50/02, although this improvement is not substantial.

In contrast, the incorporation of [Cho][Glu] proved to be more promising. This IL allowed for a much more pronounced enhancement in drug release for the three drugs (**E_Caf_**, **E_SA_**, and **E_Rut_**).

Specifically, for the more hydrophilic caffeine, the inclusion of [Cho][Glu] alone (Figure 7, **E_Caf_**) led to an increase in drug release of above 75% (from 2% to 78%) compared to the batch containing only Dynasan^®^ 118 (**A_Caf_**). Additionally, compared with the results discussed for sucrose and Gelucire^®^ 50/02, [Cho][Glu] allowed for an enhancement in drug release of about 60% (from 14% to 78%) compared to the batch containing sucrose (**C_Caf_**) and an enhancement of more than 20% (from 55% to 78%) compared to the implants containing Gelucire^®^ 50/02 (**B_Caf_**). This demonstrates that [Cho][Glu] is a better caffeine release promotor than sucrose and Gelucire^®^ 50/02.

For the implants containing the more lipophilic drugs, salicylic acid and rutin, an upper drug release of about 95% was obtained when [Cho][Glu] was incorporated (**E_SA_** and **E_Rut_**). This denotes an increase of more than 70% when compared to the batches containing only Dynasan^®^ 118 (**A_SA_** and **A_Rut_**, Figure 7). Additionally, compared to the implants with Dynasan^®^ 118 and sucrose (**C_SA_** and **C_Rut_**, Figure 5) a substantial increase was also observed (above 60%).

It is also noteworthy that in contrast to the implants containing Gelucire^®^ 50/02 (**B_SA_** and **B_Rut_**) or Gelucire^®^ 50/02 combined with the ILs (**G_SA_** and **G_Rut,_** and **F_SA_** and **F_Rut_**), the inclusion of [Cho][Glu] alone (**D_SA_** and **D_Rut_,** and **E_SA_** and **E_Rut_**) allowed for a more controlled drug release profile, similar to what was observed for [Cho][Phe].

Bearing all this in mind, the inclusion of [Cho][Glu] seems to be particularly advantageous as it not only leads to a high drug release but also this release occurs in a more controlled manner over time (throughout 140 days for caffeine and 80 days for salicylic acid and rutin). This result is aligned with what is desirable for controlled release systems as not only a long-term drug release must be attained but also this release should be as high as possible to ensure therapeutically effective plasma drug concentration levels. The improved drug release may be due to the ability of ILs to be miscible with a wide variety of solvents and solutes [21], and due to the fact that they are known to enhance drug solubility, namely of the studied compounds [13,15], which may facilitate the drug release.

In terms of the water content and lipidic erosion, generally no considerable differences were observed between the implants with and without ILs, revealing that the ILs do not seem to have much impact on these parameters. Moreover, it should be noted that our results for Dynasan^®^ 118 are consistent with a previous study [45] that also presented low values of water content and erosion for Dynasan^®^ 118 implants containing theophylline (a member of the xanthine family such as caffeine) after seven days at 37 °C in a phosphate buffer 7.4.

In terms of the drug release mechanism, it has been described that this aspect may be controlled by water or drug diffusion [45]. Our results seem to suggest that for the developed implants, drug diffusion is likely more relevant than water diffusion as for all the studied compositions, a clear difference in drug release was observed depending on the type of drug incorporated with the more lipophilic drugs presenting a higher diffusion rate. This is likely due to a higher affinity with the lipophilic implant matrix. Conversely, the water content studies did not demonstrate clear differences between the implants, thus indicating that this parameter possibly has a lower impact on the release mechanism.

#### 3.2.3. Atomic Force Microscopy (AFM)

**Figure 8:**
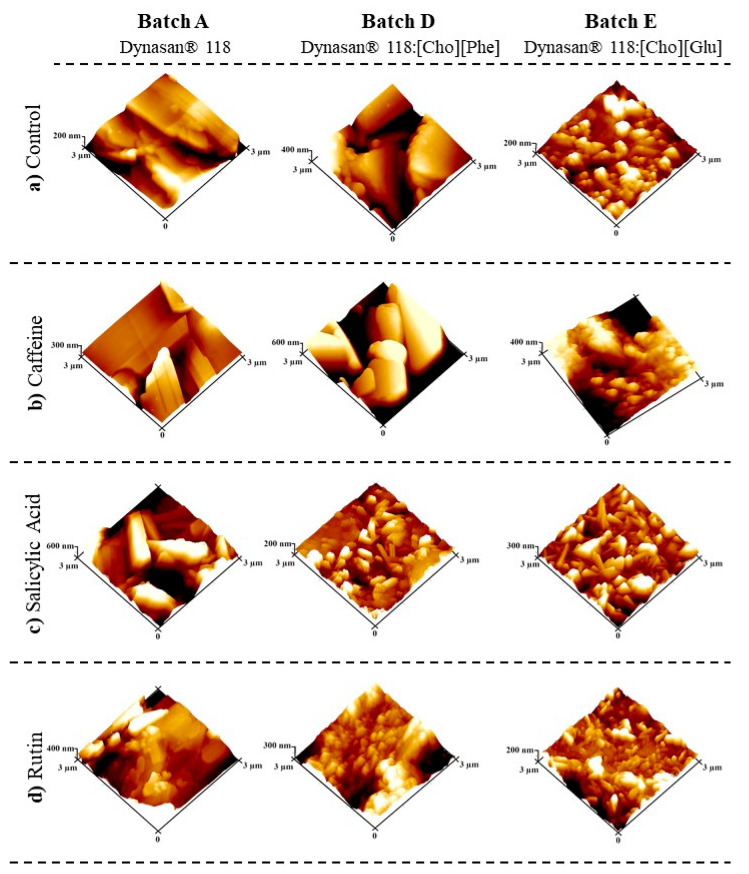
AFM images (3.0 × 3.0 μm^2^) of the implants containing only Dynasan^®^ 118 (**Batch A**), Dynasan^®^ 118 and [Cho][Phe] (**Batch D**), and Dynasan^®^ 118 and [Cho][Glu] (**Batch E**). The denoted batches were studied in the absence of drugs (**a**-**control**) or in the presence of caffeine (**b**), salicylic acid (**c**), or rutin (**d**).

Considering that the isolated incorporation of ILs seems to be the better strategy, AFM images were captured from the Dynasan^®^ 118 implants containing either the [Cho][Phe] or the [Cho][Glu] and each drug (**D_Caf_**, **D_SA_**, and **D_Rut_,** and **E_Caf_**, **E_SA_**, and **E_Rut_**), and from the respective controls with and without each drug (**A_Caf_**, **A_SA_**, and **A_Rut_,** and **A_0_**, **D_0_**, and **E_0_**). Figure 8 displays representative images of each of these implants.

The AFM technique is quite helpful to analyze surface topography and roughness through three-dimensional imaging. Interestingly, the results demonstrated that the implants containing the ILs presented a more wrinkled surface compared to the implants without an IL. Nonetheless, this wrinkling was much more prominent in the presence of [Cho][Glu], independently of the incorporated drug which is consistent with the higher drug release observed for the implants containing this IL. This result may be explained by considering that wrinkling allows for a higher surface area and may consequently lead to an increased drug release. Thus, the obtained wrinkling may also be a determinant driving force that impacts drug release in addition to drug diffusion. In the presence of each active, it was also possible to observe that the size of the crystalline structures was smaller for the implants containing the ILs, demonstrating that the ILs promote a better distribution of the drugs. Furthermore, designing wrinkle delivery systems has been considered a valuable strategy to improve release and in consequence, there has been a growing interest in designing such delivery systems [46]. Hence, this result presents a new and valuable functionality of ILs when incorporated in lipid-implants.

In terms of surface functionalization, ILs have been used for this purpose in nanoparticles [47,48] due to the effect of various intermolecular interactions between the ILs and the components present in the delivery systems that are responsible for the singular distribution of various solutes within ILs, in comparison to other solvents. This may explain the impact of the ILs on the wrinkling observed for the developed implants.

It is the ionic nature of the ILs in addition to their heterogenous structure that provides them a unique combination of strong Coulombic interactions, van der Waals, inductive and dispersion interactions, and hydrogen bond interactions [47]. For instance, it has been described that the anions present in the ILs may have a greater ability to form hydrogen bonds with drug molecules [49] and thus lead to improved drug solubility [15]. This interaction may justify both the improved drug release into the aqueous medium and the differences observed between the two studied ILs that differ only in terms of the anion present.

Thus, our results demonstrate that including ILs into lipidic implants, particularly the studied [Cho][Glu], may be a quite advantageous strategy. Specifically, ILs proved to be a talented material that improves the performance of the developed systems through a multipurposed functionality.

## 4. Conclusions

Lipidic implants may be a quite advantageous type of drug delivery system by allowing for a controlled, sustained, and localized delivery. Ensuring that these systems are produced in an easy and efficient manner that allows content and drug uniformity while achieving the desired release profile is fundamental.

Hence, this study had multiple aims including improving the preparation procedure and evaluating the influence of different compositions on this procedure, in addition to their impact on the performance of the developed implants. To achieve this, implants containing Dynasan^®^ 118 and a variable composition in terms of other constituents were prepared. Namely, Gelucire^®^ 50/02, sucrose, and two biobased ILs, [Cho][Phe] or [Cho][Glu], were included in the developed implants, either alone or in different combinations.

A modified and improved fusion and melting method was described herein that allowed for a faster and easier procedure with less material loss. Moreover, the implants containing ionic liquids proved to be much easier to blend both in the absence or presence of the studied drugs, demonstrating that the ILs further improved the preparation of the developed systems.

To evaluate whether the preparation technique and the various excipients used would lead to a homogeneous distribution, the lipophilic dye, Sudan III, and the hydrophilic dye, Methylene Blue, were incorporated into the various implants with different compositions. The prepared implants presented a uniform distribution of each dye, indicating that the preparation technique leads to an even incorporation of both lipophilic and hydrophilic substances. Following this, the three studied drugs, caffeine, salicylic acid, and rutin were included in the developed systems and the drug content was assessed. All implants exhibited a drug content superior to 95% without statistical differences between them.

It was also clear that the type of excipient included in the formulations had a considerable impact on the performance of the implants. In fact, for the implants containing sucrose, generally the drug release was lower compared to the implants containing Gelucire^®^ or ILs. Nonetheless, in the presence of Gelucire^®^, the implants containing the lipophilic drugs salicylic acid or rutin presented an immediate drug release which is not desirable. In contrast, the incorporation of each IL alone, particularly [Cho][Glu], proved to be the better choice in terms of performance. In fact, the incorporation of these biobased materials led to implants acquiring a more wrinkled surface, allowing for a higher and more suitable release profile for almost 3 months without having impact on the water content or lipidic erosion. The results suggest that drug diffusion and surface wrinkling may be the key factors for drug release.

Hence, the studied ILs proved to be multitalented materials by demonstrating various functionalities when included in lipidic implants. Namely, at non-toxic concentrations, these biobased compounds allowed for an easier formulation of the implants and facilitated the incorporation of both lipophilic and hydrophilic drugs, while allowing to alter the surface properties of the implants and refining the drug release, especially in the case of [Cho][Glu].

## Figures and Tables

**Table 1:** Composition, % (*w*/*w*), of all the Dynasan^®^ 118 lipidic implants containing variable compositions of Gelucire^®^ 50/02, sucrose, and each IL ([Cho][Phe] or [Cho][Glu]). Implants (A–I) were prepared without (controls) or in the presence of each dye (methylene blue or sudan III) or each drug (caffeine, salicylic acid, or rutin).

Drug	Formulations		% *w*/*w*			
		Dynasan^®^ 118	Gelucire^®^ 50/02	Sucrose	[Cho][Phe]	[Cho][Glu]
Controls (without drug)	A_0_	100	-	-	-	-
	B_0_	90	10	-	-	-
	C_0_	90	-	10	-	-
	D_0_	99.8	-	-	0.2	-
	E_0_	99.8	-	-	-	0.2
	F_0_	89.8	10	-	0.2	-
	G_0_	89.8	10	-	-	0.2
	H_0_	89.8	-	10	0.2	-
	I_0_	89.8	-	10	-	0.2
Dye2.5% *w*/*w* (Methylene blue orSudan III)	A_Dye_	97.5	-	-	-	-
	B_Dye_	87.5	10	-	-	-
	C_Dye_	87.5	-	10	-	-
	D_Dye_	97.3	-	-	0.2	-
	E_Dye_	97.3	-	-	-	0.2
	F_Dye_	87.3	10	-	0.2	-
	G_Dye_	87.3	10	-	-	0.2
	H_Dye_	87.3	-	10	0.2	-
	I_Dye_	87.3	-	10	-	0.2
Drug 10% *w*/*w* (caffeine, salicylic acid, or rutin)	A_Drug_	90	-	-	-	-
	B_Drug_	80	10	-	-	-
	C_Drug_	80	-	10	-	-
	D_Drug_	89.8	-	-	0.2	-
	E_Drug_	89.8	-	-	-	0.2
	F_Drug_	79.8	10	-	0.2	-
	G_Drug_	79.8	10	-	-	0.2
	H_Drug_	79.8	-	10	0.2	-
	I_Drug_	79.8	-	10	-	0.2

## References

[1] Shi Y., Li L.C. (2005). Current advances in sustained-release systems for parenteral drug delivery. Expert Opin. Drug Deliv..

[2] García-Estrada P., García-Bon M.A., López-Naranjo E.J., Basaldúa-Pérez D.N., Santos A., Navarro-Partida J. (2021). Polymeric Implants for the Treatment of Intraocular Eye Diseases: Trends in Biodegradable and Non-Biodegradable Materials. Pharmaceutics.

[3] Kreye F., Siepmann F., Siepmann J. (2008). Lipid implants as drug delivery systems. Expert Opin. Drug Deliv..

[4] Koennings S., Garcion E., Faisant N., Menei P., Benoit J.P., Goepferich A. (2006). In vitro investigation of lipid implants as a controlled release system for interleukin-18. Int. J. Pharm..

[5] Kumar A., Pillai J. (2018). Implantable drug delivery systems: An overview. Nanostructures Eng. Cells Tissues Organs.

[6] Kreye F., Siepmann F., Willart J.F., Descamps M., Siepmann J. (2011). Drug release mechanisms of cast lipid implants. Eur. J. Pharm. Biopharm..

[7] Kreye F., Siepmann F., Zimmer A., Willart J.F., Descamps M., Siepmann J. (2011). Controlled release implants based on cast lipid blends. Eur. J. Pharm. Sci..

[8] Guse C., Koennings S., Kreye F., Siepmann F., Goepferich A., Siepmann J. (2006). Drug release from lipid-based implants: Elucidation of the underlying mass transport mechanisms. Int. J. Pharm..

[9] Nicolai M., Amaral V., Antunes C., Shuwisitkul D., Portugal Mota J. (2014). Lipid-Based Implants: Impact of Formulation Parameters. Adv. Mater. Res..

[10] Koennings S., Sapin A., Blunk T., Menei P., Goepferich A. (2007). Towards controlled release of BDNF—Manufacturing strategies for protein-loaded lipid implants and biocompatibility evaluation in the brain. J. Control. Release.

[11] Gouveia W., Jorge T.F., Martins S., Meireles M., Carolino M., Cruz C., Almeida T.V., Araújo M.E.M. (2014). Toxicity of ionic liquids prepared from biomaterials. Chemosphere.

[12] Caparica R., Júlio A., Baby A.R., Eduarda M.E.A., Fernandes A.S., Costa J.G., Santos de Almeida T. (2018). Choline-Amino Acid Ionic Liquids as Green Functional Excipients to Enhance Drug Solubility. Pharmaceutics.

[13] de Almeida T.S., Júlio A., Saraiva N., Fernandes A.S., Araújo M.E.M., Baby A.R., Rosado C., Mota J.P. (2017). Choline- versus imidazole-based ionic liquids as functional ingredients in topical delivery systems: Cytotoxicity, solubility, and skin permeation studies. Drug Dev. Ind. Pharm..

[14] Czekanski L., de Almeida T.S., Portugal Mota J., Rijo P., Araújo M.E.M. (2014). Synthesis of benzoazole ionic liquids and evaluation of their antimicrobial activity. J. Biomed. Biopharm. Res..

[15] Caparica R., Júlio A., Fernandes F., Araújo M.E.M., Costa J.G., de Almeida T.S. (2021). Upgrading the topical delivery of poorly soluble drugs using ionic liquids as a versatile tool. Int. J. Mol. Sci..

[16] Silva A.T., Teixeira C., Marques E.F., Prudêncio C., Gomes P., Ferraz R. (2021). Surfing the Third Wave of Ionic Liquids: A Brief Review on the Role of Surface-Active Ionic Liquids in Drug Development and Delivery. ChemMedChem.

[17] Shmukler L.E., Fedorova I.V., Fadeeva Y.A., Safonova L.P. (2021). The physicochemical properties and structure of alkylammonium protic ionic liquids of RnH4-nNX (n = 1–3) family. A mini–review. J. Mol. Liq..

[18] Pedro S.N., Freire C.S.R., Silvestre A.J.D., Freire M.G. (2020). The role of ionic liquids in the pharmaceutical field: An overview of relevant applications. Int. J. Mol. Sci..

[19] Marrucho I.M., Branco L.C., Rebelo L.P.N. (2014). Ionic liquids in pharmaceutical applications. Annu. Rev. Chem. Biomol. Eng..

[20] Mitkare S.S., Lakhane K.G., Kokulwar P.U. (2013). Ionic liquids: Novel Applications in Drug Delivery. Res. J. Pharm. Technol..

[21] Sidat Z., Marimuthu T., Kumar P., du Toit L.C., Kondiah P.P.D., Choonara Y.E., Pillay V. (2019). Ionic liquids as potential and synergistic permeation enhancers for transdermal drug delivery. Pharmaceutics.

[22] Caparica R., Júlio A., Araújo M.E.M., Baby A.R., Fonte P., Costa J.G., de Almeida T.S. (2020). Anticancer activity of rutin and its combination with ionic liquids on renal cells. Biomolecules.

[23] Shamshina J.L., Barber P.S., Rogers R.D. (2013). Ionic liquids in drug delivery. Expert Opin. Drug Deliv..

[24] Ferraz R., Branco L.C., Prudêncio C., Noronha J.P., Petrovski Ž. (2011). Ionic liquids as active pharmaceutical ingredients. ChemMedChem.

[25] Dobler D., Schmidts T., Klingenhoefer I., Runkel F. (2012). Ionic liquids as ingredients in topical drug delivery systems. Int. J. Pharm..

[26] Zech O., Thomaier S., Bauduin P., Rück T., Touraud D., Kunz W. (2009). Microemulsions with an ionic liquid surfactant and room temperature ionic liquids as polar pseudo-phase. J. Phys. Chem. B.

[27] Zhu W., Guo C., Yu A., Gao Y., Cao F., Zhai G. (2009). Microemulsion-based hydrogel formulation of penciclovir for topical delivery. Int. J. Pharm..

[28] Júlio A., Costa Lima S.A., Reis S., de Almeida T.S., Fonte P. (2019). Development of ionic liquid-polymer nanoparticle hybrid systems for delivery of poorly soluble drugs. J. Drug Deliv. Sci. Technol..

[29] Júlio A., Caparica R., Costa Lima S.A., Fernandes A.S., Rosado C., Prazeres D.M.F., Reis S., de Almeida T.S., Fonte P. (2019). Ionic liquid-polymer nanoparticle hybrid systems as new tools to deliver poorly soluble drugs. Nanomaterials.

[30] de Almeida T.S., Júlio A., Mota J.P., Rijo P., Reis C.P. (2017). An emerging integration between ionic liquids ans nanotechnology: General uses and future prospects in drug delivery. Ther. Deliv..

[31] Luo Q., Pentzer E. (2020). Encapsulation of Ionic Liquids for Tailored Applications. ACS Appl. Mater. Interfaces.

[32] Herman A., Herman A.P. (2012). Caffeine’s mechanisms of action and its cosmetic use. Ski. Pharmacol. Physiol..

[33] Lupi O., Semenovitch I.J., Treu C., Bottino D., Bouskela E. (2007). Evaluation of the effects of caffeine in the microcirculation and edema on thighs and buttocks using the orthogonal polarization spectral imaging and clinical parameters. J. Cosmet. Dermatol..

[34] Velasco M.V.R., Tano C.N., Machado-Santelli G.M., Consiglieri V.O., Kaneko T.M., Baby A.R. (2008). Effects of caffeine and siloxanetriol alginate caffeine, as anticellulite agents, on fatty tissue: Histological evaluation. J. Cosmet. Dermatol..

[35] Choi H.W., Tian M., Manohar M., Harraz M.M., Park S.W., Schroeder F.C., Snyder S.H., Klessig D.F. (2015). Human GAPDH is a target of aspirin’s primary metabolite salicylic acid and its derivatives. PLoS ONE.

[36] Ferré S. (2016). Mechanisms of the psychostimulant effects of caffeine: Implications for substance use disorders. Psychopharmacology.

[37] Ganeshpurkar A., Saluja A.K. (2017). The Pharmacological Potential of Rutin. Saudi Pharm. J..

[38] Kolahdouzan M., Hamadeh M.J. (2017). The neuroprotective effects of caffeine in neurodegenerative diseases. CNS Neurosci. Ther..

[39] Sharma S., Ali A., Ali J., Sahni J.K., Baboota S. (2013). Rutin: Therapeutic potential and recent advances in drug delivery. Expert Opin. Investig. Drugs.

[40] Bari H. (2010). A prolonged release parenteral drug delivery system—An overview. Int. J. Pharm. Sci. Rev. Res..

[41] Pezzini B.R., Silva M.A.S., Ferraz H.G. (2007). Formas farmacêuticas sólidas orais de liberação prolongada: Sistemas monolíticos e multiparticulados. Rev. Bras. Ciencias Farm.

[42] Gowda D.V., Aravind Ram A.S., Venkatesh M.P., Khan M.S. (2012). Development and evaluation of clozapine pellets for controlled release. Int. J. Res. Ayurveda Pharm..

[43] Asmus L.R., Gurny R., Möller M. (2011). Solutions for lipophilic drugs: A biodegradable polymer acting as solvent, matrix, and carrier to solve drug delivery issues. Int. J. Artif. Organs.

[44] Hyun D.C. (2015). A Polymeric Bowl for Multi-Agent Delivery. Macromol. Rapid Commun..

[45] Kreye F., Siepmann F., Siepmann J. (2011). Drug release mechanisms of compressed lipid implants. Int. J. Pharm..

[46] Christian P., Ehmann H.M.A., Werzer O., Coclite A.M. (2016). Wrinkle formation in a polymeric drug coating deposited via initiated chemical vapor deposition. Soft Matter.

[47] He Z., Alexandridis P. (2015). Nanoparticles in ionic liquids: Interactions and organization. Phys. Chem. Chem. Phys..

[48] Patil A.B., Bhanage B.M. (2014). Shape selectivity using ionic liquids for the preparation of silver and silver sulphide nanomaterials. Phys. Chem. Chem. Phys..

[49] Huang W., Wu X., Qi J., Zhu Q., Wu W., Lu Y., Chen Z. (2020). Ionic liquids: Green and tailor-made solvents in drug delivery. Drug Discov. Today.

